# Arsenite exposure inhibits the erythroid differentiation of human hematopoietic progenitor CD34^+^ cells and causes decreased levels of hemoglobin

**DOI:** 10.1038/s41598-021-01643-2

**Published:** 2021-11-11

**Authors:** Guanghua Wan, Sebastian Medina, Haikun Zhang, Rong Pan, Xixi Zhou, Alicia M. Bolt, Li Luo, Scott W. Burchiel, Ke Jian Liu

**Affiliations:** 1 Department of Pharmaceutical Sciences, The University of New Mexico College of Pharmacy, Albuquerque, NM 87131 USA; 2 Department of Biology, New Mexico Highlands University, Las Vegas, NM 87701 USA; 3 Division of Epidemiology, Biostatistics and Preventive Medicine at the University of New Mexico, Albuquerque, NM 87131 USA

**Keywords:** Anaemia, Haematopoietic stem cells, Environmental impact

## Abstract

Arsenic exposure poses numerous threats to human health. Our previous work in mice has shown that arsenic causes anemia by inhibiting erythropoiesis. However, the impacts of arsenic exposure on human erythropoiesis remain largely unclear. We report here that low-dose arsenic exposure inhibits the erythroid differentiation of human hematopoietic progenitor cells (HPCs). The impacts of arsenic (in the form of arsenite; As^3+^) on red blood cell (RBC) development was evaluated using a long-term culture of normal human bone marrow CD34^+^-HPCs stimulated in vitro to undergo erythropoiesis. Over the time course studied, we analyzed the expression of the cell surface antigens CD34, CD71 and CD235a, which are markers commonly used to monitor the progression of HPCs through the stages of erythropoiesis. Simultaneously, we measured hemoglobin content, which is an important criterion used clinically for diagnosing anemia. As compared to control, low-dose As^3+^ exposure (100 nM and 500 nM) inhibited the expansion of CD34^+^-HPCs over the time course investigated; decreased the number of committed erythroid progenitors (BFU-E and CFU-E) and erythroblast differentiation in the subsequent stages; and caused a reduction of hemoglobin content. These findings demonstrate that low-dose arsenic exposure impairs human erythropoiesis, likely by combined effects on various stages of RBC formation.

## Introduction

Erythropoiesis is a highly regulated and dynamic process that occurs primarily in the bone marrow (BM) of adult humans^1,2^. As shown in the schematic diagram of normal red blood cell (RBC) production (Supplementary Fig. S1), the process starts from pluripotent hematopoietic stem cells (HSCs), which differentiate to common myeloid progenitors (CMPs) and then megakaryocyte/erythroid progenitors (MEPs). MEPs differentiate to the committed erythroid progenitor cell stages upon stimulation with growth factors, including erythropoietin, stem cell factor, and interleukin 3^2,3^. The earliest detectable committed erythroid progenitors are burst-forming unit erythroid cells (BFU-E), which further differentiate into colony-forming unit erythroid cells (CFU-E). CFU-E progenitors are highly proliferative and divide 3–5 times over 2–3 days before differentiating to pro-erythroblasts (ProE). The ProE cells undergo further differentiation to the basophilic (BasoE), polychromatic (PolyE) and orthochromatic (OrthoE) erythroblast stages, prior to enucleation and exiting from the bone marrow as reticulocytes (Retic), which complete maturation in systemic circulation to yield full functional erythrocytes^2,3^.

Throughout erythropoiesis, erythroid progenitor cells undergo many substantial changes, including a decrease in cell volume/size, a massive increase in hemoglobin production, and chromatin condensation, leading up to their enucleation and expulsion of cellular organelles^1–4^. These changes are paralleled by the dynamic changes of important cell surface antigens, including CD34, CD71, and CD235a, which are critical for proliferation, differentiation and maturation^5–7^. Expression of these cell surface proteins is dependent on the stages of erythropoiesis (Supplementary Fig. S1). CD34 is highly expressed in HPCs at the initial stage, gradually decreased along the proliferation and erythroid differentiation and then disappeared at the CFU-E stage^5–7^. CD71 increases and co-expresses with CD34 at the early stages of erythropoiesis and is highly expressed in CFU-E and proerythroblasts (ProE). CD71 is gradually downregulated from the late stage of erythroblasts (BasoE and polyE) to the terminal differentiation stage (OrthoE) and is absent from the mature red blood cells (RBCs)^5–7^. CD71 co-expresses with CD235a in human erythroblasts. CD235a expression is low in ProE, but its expression level increases thereafter along the continuum of erythroblasts differentiation, reaching maximum levels in final maturated erythrocytes^5^. The stage-dependent appearance, disappearance and expression levels of these cell surface antigens is tightly controlled under normal conditions of erythropoiesis^2,3,5–7^, and thus they are used as stage-specific identifiers in the study of erythropoiesis.

Dysfunction of erythropoiesis can be caused by many environmental factors including arsenic exposure^8–12^. Arsenic is a naturally occurring element that widely exists in the environment and is a common contaminant in soils, air, food and water^13–15^. Chronic, low-level arsenic exposures result in various adverse health effects^16–20^. Epidemiological studies provide strong evidence of an increased incidence of anemia in people who are chronically exposed to arsenic in drinking water at concentrations exceeding the WHO safety standard of 10 µg/L (ppb)^15,21–25^.

Anemia is a hematological disorder classified by too few RBCs in circulation and/or by a lower-than-normal production of hemoglobin (Hb)^26,27^. Anemia can be caused by decreased RBC production, increased RBC destruction, or other circumstances, such as blood loss^28–30^. Arsenic-induced destruction of RBCs is mainly caused by cytotoxicity of relatively high arsenic doses^8–10^. The suppressive effects of arsenic exposure, at low environmentally relevant concentrations, on erythropoiesis have also been reported in adult male mice^11,12^. However, the effects of low-dose arsenic exposure on human erythropoiesis remain largely unclear.

Several previous reports suggest that arsenic exposures impair certain stages of erythropoiesis^11,12^. Morse et al. showed the effects of arsenic on erythropoietin-induced erythroid differentiation in mice, which revealed a significant inhibitory effect on early-stage, proliferating nucleated erythroid precursor cells but not more mature, non-proliferating nucleated erythroid cells^11^. Additionally, previous in vitro and in vivo studies from our laboratory show that exposure to environmentally relevant levels of arsenite (As^3+^; 100 ppb and 500 ppb) suppress the development of erythroid progenitors in the bone marrow resulting in the development of anemia in adult male mice^12^. However, little is known about whether the disruption of RBC maturation observed in rodents is relevant to human erythropoiesis.

In this study, we investigated the inhibitory effects of arsenic on human erythropoiesis by examining how arsenic exposure alters the dynamic changes of important cell surface markers (i.e., CD34, CD71 and CD235a) and whether arsenic exposure affects Hb production. We selected human bone marrow CD34^+^ hematopoietic progenitor cells (HPCs) as our experimental model because CD34^+^-HPCs are multi-potential, and depending on the stimulus provided, can fully reconstitute the hematopoietic system, including RBCs^31–37^. By maintaining CD34^+^-HPCs in suitable medium supplemented with erythroid expansion factors, the overall process of human erythropoiesis can be modeled in vitro^31–37^. The findings from the present study provide novel information regarding the specific stages of erythropoiesis disrupted by arsenic exposure using a human relevant experimental model system.

## Results

### As^3+^ exposure inhibits proliferation and decreases cell viability at late stages of erythroid development during the erythroid expansion of CD34^+^-HPCs

**Figure 1:**
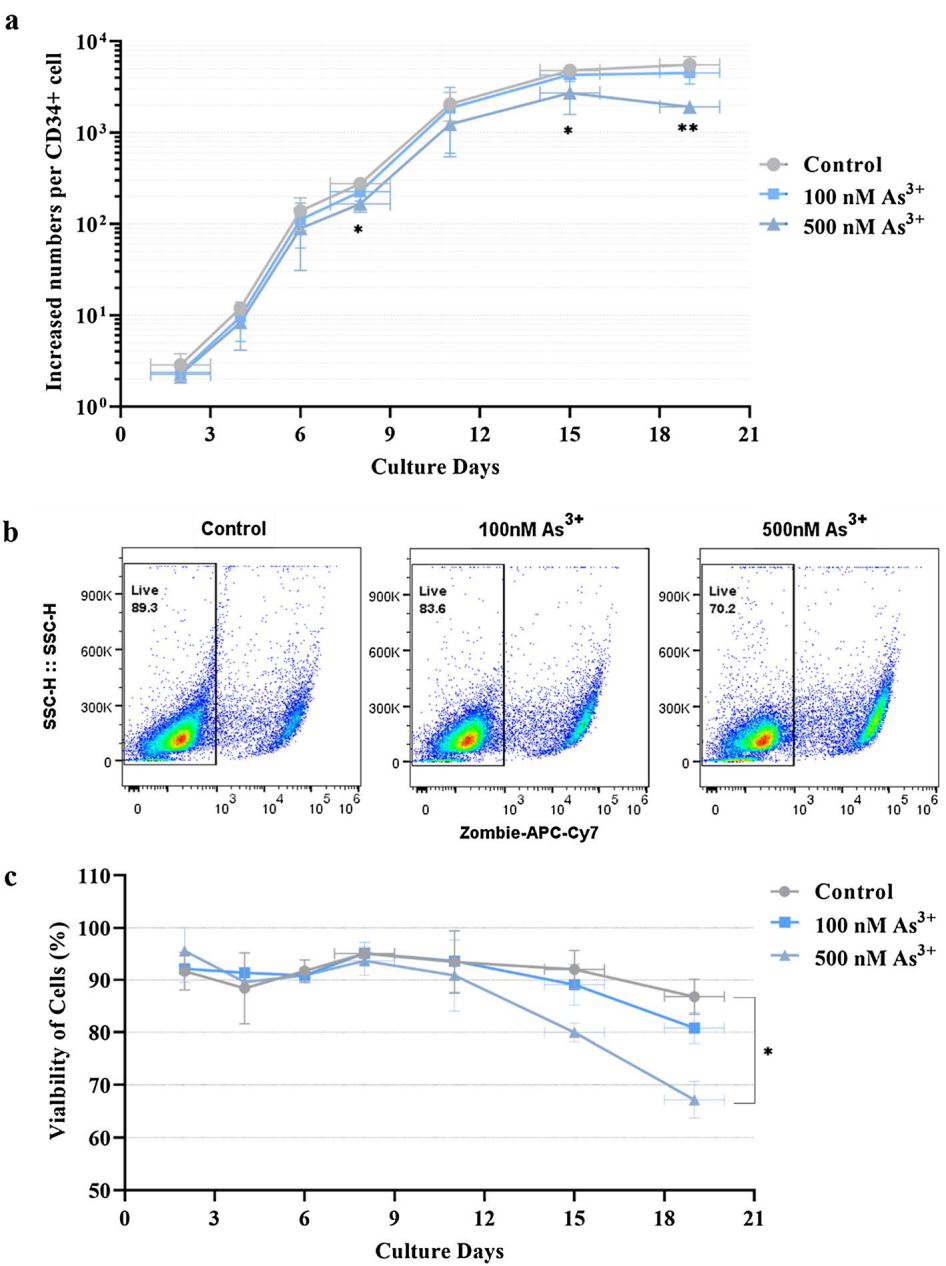
As^3+^ exposure decreases cell growth during the erythroid expansion of human CD34^+^-HPCs. Human Bone Marrow CD34^+^-HPCs at an initial concentration of 1 × 10^4^ cells/mL were cultured for the indicated days in erythroid expansion medium with or without As^3+^. (**a**) Cell growth was recorded as the counts per initial CD34^+^ cells in the presence of 0 (Control), 100, or 500 nM As^3+^ at each of the indicated time points. (**b**) Representative flow cytometry plots showing cell viability on day 18. (**c**) Summary of cell viability results over the time course investigated. Data are expressed as summarized mean ± SD from three donors, *n* = 3 replicates/donor/group, *(*p* < 0.05), and **(*p* < 0.01) in one-way ANOVA, followed by Tukey’s post hoc test between the groups. * and ** indicate statistically significant differences between control and 500 nM As^3+^.

Maintaining the survival and proliferation of erythroid precursor cells is vital for normal erythropoiesis^38^. The proliferation and expansion of erythroid precursor cells mainly occurs at the early stages of erythropoiesis (BFU-E and CFU-E)^1,38^. To investigate whether As^3+^ exposure inhibits the differentiation of early erythroid progenitors, we measured cell proliferation by counting total cell numbers throughout the 20-day differentiation time course. The numbers of CD34^+^-HPCs were rapidly amplified from day 2 to 15, while no significant changes in growth were seen after day 15 (Fig. 1a). Importantly, after day 15, the total numbers of cells in the 500 nM As^3+^ group were significantly reduced compared to the control (Fig. 1a). These data suggest that 500 nM As^3+^ exposure inhibits the erythroid expansion of CD34^+^-HPCs. Consistent results, displaying the inhibitory effects of As^3+^ exposure on cell growth were obtained using CD34^+^-HPCs from three independent donors (Supplementary Fig. S2).

To determine whether the decrease of cell numbers was due to As^3+^-induced cytotoxicity, we measured the viable cell population across the time course using flow cytometry by staining with an amine reactive fluorescent viability dye (Fig. 1b,c). Representative flow cytometry plots showing the gating criteria used to define live cells is shown in Fig. 1b. As^3+^ exposure did not alter cell viability at early stages of erythroid differentiation (days 2 to 16); but significantly decreased cell viability when exposed to 500 nM As^3+^ at later stages of differentiation and maturation (days 16 to 20) (Fig. 1c).

These data suggest that the As^3+^-induced suppression of proliferation is not substantially mediated by cytotoxicity in the early time points. However, at later culture days (day 16–20), the decrease in cell numbers is most likely due to cell death, especially with 500 nM As^3+^.

### As^3+^ exposure attenuates BFU-E and CFU-E colony formation

**Figure 2:**
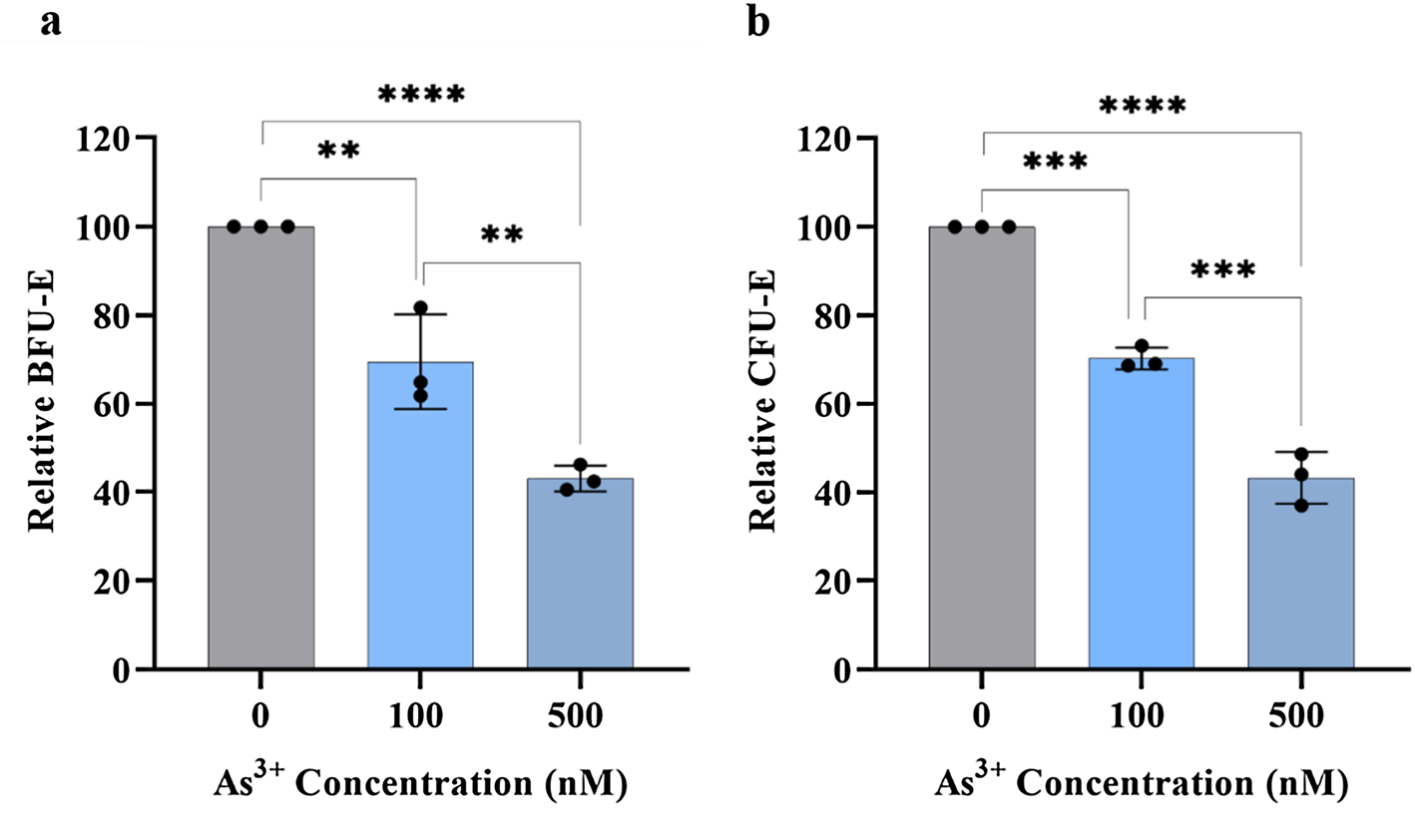
As^3+^ exposure decreases BFU-E and CFU-E colony formation. Five-hundred human bone marrow CD34^+^-HPCs were mixed with 1.1 ml of MethoCult™ medium containing 0, 100 nM, or 500 nM As^3+^. After 14–16 days of incubation, colonies were scored under an inverted light microscope. The relative number of BFU-E and CFU-E colonies for each group were calculated as described in the methods section. (**a**) Relative numbers of BFU-E colonies, (**b**) relative numbers of CFU-Es colonies. Data are expressed as mean ± SD, *n* = 3 replicates/group, **(*p* < 0.01), ***(*p* < 0.001), and ****(*p* < 0.0001) in one-way ANOVA, followed by Tukey’s post hoc test between the groups.

CFUs assays were used to monitor the formation of BFU-E and CFU-E colonies. The colony forming ability represents how many progenitors are present in a given population of cells, is widely used to estimate the ability of proliferation and differentiation of individual HPCs within a sample^3,39^. As described in the methods section, we measured the ratio of BFU-E or CFU-E numbers relative to the initial input cell number to evaluate the effects of low dose As^3+^ exposure on the colony forming ability of BFU-E and CFU-E cells derived from human bone marrow CD34^+^-HPCs. For each of the three donors, the comparison for the actual numbers of BFU-E and CFU-E colonies between control and As^3+^ exposed cultures are shown in Supplementary Fig. S3. The relative numbers of BFU-E and CFU-E colonies from all three individual donors were averaged and used for statistical analysis in Fig. 2. A significant dose-dependent reduction of both BFU-E and CFU-E colony formation was observed starting from concentration of As^3+^ as low as 100 nM (Fig. 2a,b). Both BFU-E and CFU-E colony formation was suppressed by approximately 60% with the 500 nM As^3+^ exposure (Fig. 2a,b).

The results from the analysis of BFU-E and CFU-E colony formation using CD34^+^-HPCs from three individual donors strongly suggests that As^3+^ exposure at low doses decreases the formation of committed erythroid progenitor cells at early stages of erythroid differentiation.

### As^3+^ exposure did not change CD34 expression, but altered CD71 and CD235a expression during the erythroid differentiation of human CD34^+^-HPCs

CD34 is a maker of the HPCs in early stage of proliferation and erythroid differentiation, CD71 is a marker of erythroid precursors, while the CD235a is a marker of erythroblasts later than CD71^33,40,41^ (Supplementary Fig. S1). Along the time course of CD34^+^ cell expansion toward erythroid differentiation (Day 2–19 ± 1), we measured the positive populations of CD34, CD71 and CD235a using flow cytometry (Supplementary Fig. S4a,b). Simultaneously, we analyzed the proportion of CD34^High^, CD71^High^, and CD235a^High^, which are the cell subsets with the highest expression levels (i.e., based on intensity of fluorescence) of CD34, CD71 and CD235a, respectively, to show the dynamic changes of these proteins during erythroid development from early-stage progenitors to late-stage erythroblasts (Supplementary Fig. S4c–e)^3^. The alteration of positive populations and the proportions of CD34^High^, CD71^High^, and CD235a^High^ were then compared between the control (unexposed) cells and the cells exposed to As^3+^.

Neither the total CD34^+^ population or the proportion of CD34^High^ cells was decreased along the time points from days 2 to 11. There were no significant changes of total CD34^+^ cells or the proportion of CD34^High^ cells between control, 100 nM, and 500 nM As^3+^ over the time course investigated (Supplementary Fig. S5). Therefore, As^3+^ exposure at low doses does not alter CD34 expression levels, nor its expected disappearance during the erythroid expansion of CD34^+^-HPCs.

**Figure 3:**
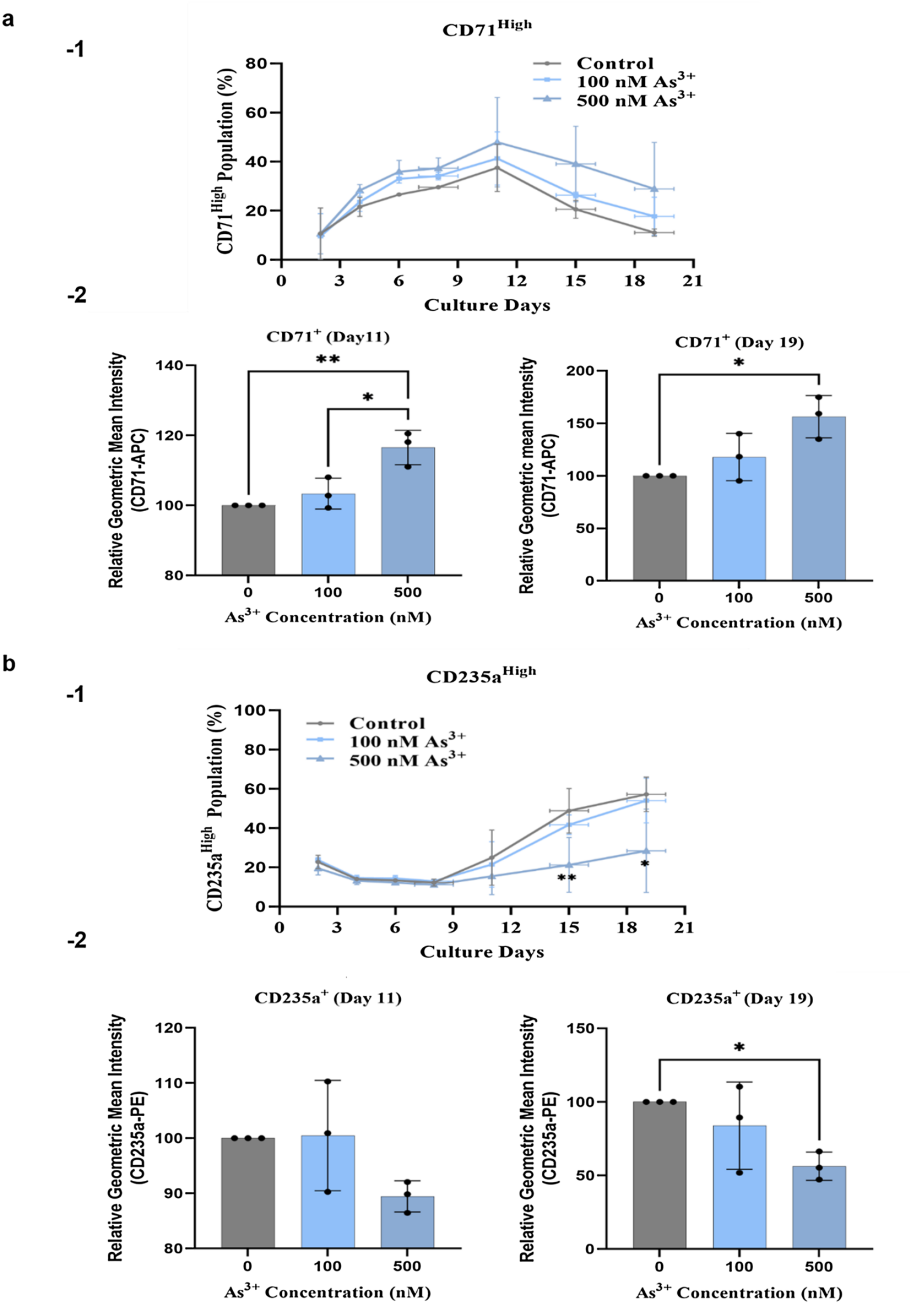
As^3+^ exposure increases CD71 signal, but decreases CD235a expression during erythroid expansion of CD34^+^ cells. Human bone marrow CD34^+^-HPCs (1 × 10^4^ cells/ml) were cultured for 2–19 (± 1) days in erythroid expansion medium with the addition of 0, or 100 nM or 500 nM As^3+^. At the time points (days 2, 4, 6, 8/9, 11, 15/16, and 19 ± 1), cells were stained with APC-conjugated antibody against CD71 (CD71-APC), and PE-conjugated antibody against CD235a (CD235a-PE) and analyzed by flow cytometry. The definitions of each cell population, including CD71^+^, CD71^High^, CD235a^+^, and CD235a^High^, are indicated in Supplementary Figs. S1 and S4. (**a-1**) As^3+^ exposure increases CD71^High^ populations throughout the time course investigated. (**a-2**) As^3+^ exposure at 500 nM significantly increases the mean fluorescence intensity of CD71^+^ cells on days 11 and 19 (± 1). (**b-1**) As^3+^ exposure (500 nM) decreases CD235a signal. (**b-2**) As^3+^ exposure (500 nM) significantly decreases mean fluorescence intensity of CD235a^+^ cells on day 11 and 19 (± 1). Data are expressed as summarized mean ± SD from three donors, *n* = 3 replicates/donor/group, *(*p* < 0.05), and **(*p* < 0.01) in one-way ANOVA, followed by Tukey’s post hoc test between the groups.

Regarding CD71, as compared to control, As^3+^ exposure did not significantly affect the total CD71^+^ cell population (Supplementary Fig. S6a-1). CD71^High^ cells were increased day-by-day reaching a top level on day 11, and thereafter gradually decreased daily (Fig. 3a-1). These CD71 expression curves (Fig. 3a-1), mirrored the dynamic changes of CD71 expression during erythropoiesis as shown in Supplementary Fig. S1. Importantly, the proportion of CD71^High^ cells was increased by As^3+^ exposure as compared to control at almost all of the tested time points during the erythroid differentiation of CD34^+^-HPCs (Fig. 3a-1, Supplementary Fig. S7a,b). Moreover, the mean intensity of CD71^+^ cells was significantly increased by As^3+^ exposure on days 11 and 19 ± 1 (Fig. 3a-2), while the mean intensity of CD7^High^ also showed an increasing trend in the presence of 500 nM As^3+^ (Supplementary Fig. S6a-2). These results indicate that As^3+^ exposure may cause an increased signal of CD71, especially at the late stages of erythroid differentiation of CD34^+^-HPCs.

As^3+^ exposure did not significantly alter the CD235a^+^ cell population (Fig. 3b-1). As expected, CD235a^High^ erythroblasts were relatively low at early days of the differentiation time course, but increased daily from days 11 to 19 ± 1 (Fig. 3b-2). This indicates that cellular CD235a content increases along the erythroblast differentiation time course, which is consistent with the expected changes in CD235a expression during human erythropoiesis (Supplementary Fig. S1). Importantly, as compared to control, the proportion of CD235a^High^ was decreased from day 11 with the 500 nM As^3+^ exposure (Fig. 3b-1), Supplementary Fig. S7c,d). The mean fluorescence intensity of CD235a^+^ cells was significantly decreased by 500 nM As^3+^ exposure on day 11 and day 19 ± 1 (Fig. 3b-2), and a decreasing trend of mean fluorescence intensity of CD235a^High^ cells was also observed with 500 nM As^3+^ (Supplementary Fig. S6b-2). These results suggest that As^3+^ exposure results in decreased erythroblast maturation, as indicated by a reduction of CD235a signal.

### As^3+^ exposure inhibits the erythroid differentiation of human CD34^+^-HPCs

**Figure 4:**
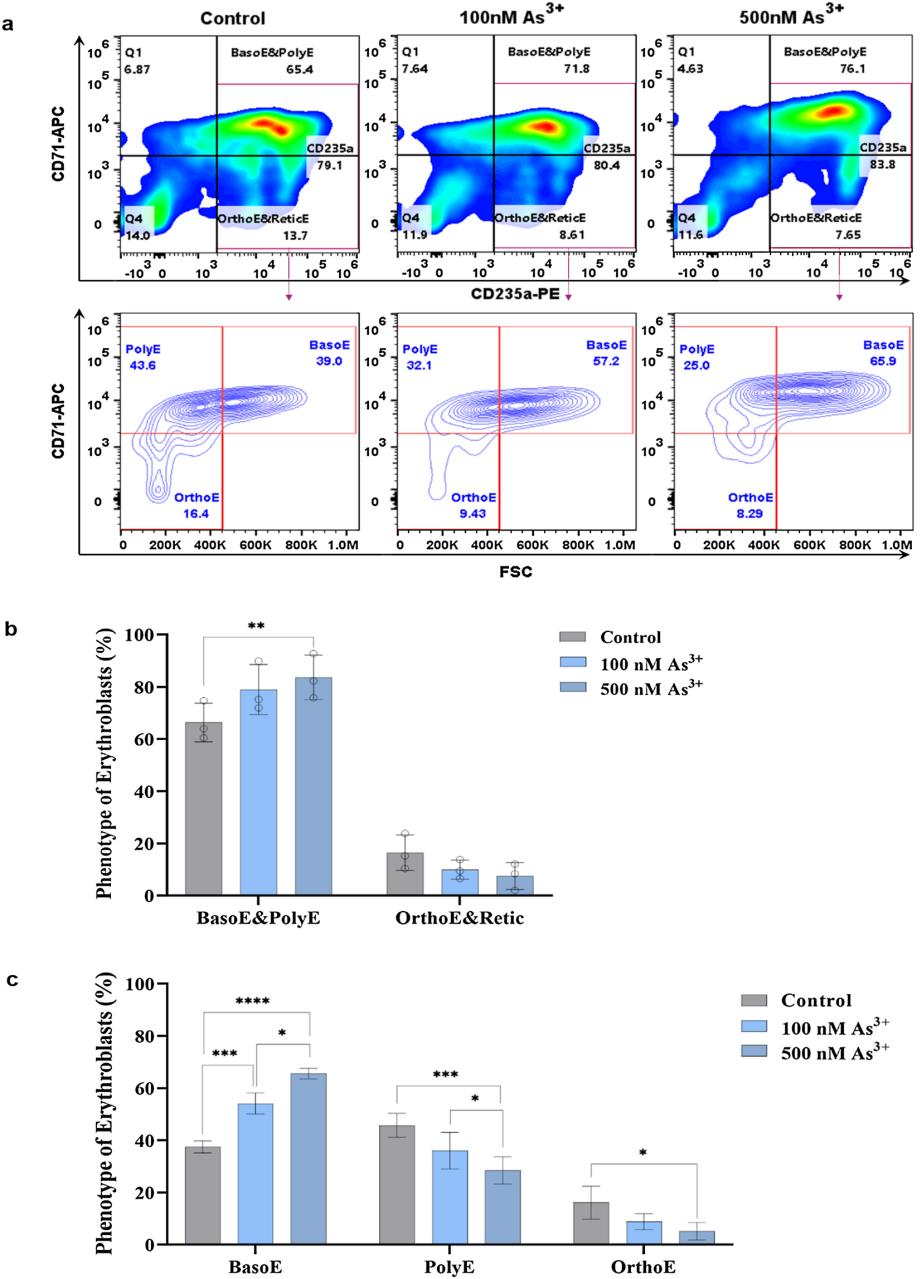
As^3+^ exposure impairs erythroblast differentiation and maturation (on day 19 ± 1). CD34^+^-HPCs (1 × 10^4^ cells/ml) were cultured for 19 (± 1) days in erythroid expansion medium in the presence of 0, 100 nM, or 500 nM As^3+^. Cells were labeled with APC-conjugated antibody against CD71 (CD71-APC) and PE-conjugated antibody against CD235a (CD235a-PE) and were analyzed by flow cytometry. (**a**) Identification of erythroblasts phenotypes were performed using two gating strategies. One is based on the plot of CD235a versus CD71 (Top-panel). Cells were distinguished as distinct subpopulations BasoE/PolyE, and OrthoE/ReticE. The second is based on plot of CD235a^+^ cells using CD71-APC versus FSC-A (Bottom-panel). By this method, CD235a^+^ cells on the top-panel can be further separated as 3 subsets: BasoE, PolyE and OrchoE. (**b**) As^3+^ exposure increases the population of less mature erythroblasts (BasoE/PolyE) and decreases the number of more mature erythroblasts (OrthoE/ReticE) on Day 19 ± 1 of the erythroid expansion of human CD34^+^ cells. (**c**) As^3+^ exposure increases the population of less mature erythroblasts, BasoE and decreases the number of more mature erythroblasts, OrthoE. Data are expressed as mean ± SD, *n* = 3 replicates/group, *(*p* < 0.05), **(*p* < 0.01), ***(*p* < 0.001), and ****(*p* < 0.0001) in one-way ANOVA, followed by Tukey’s post hoc test between the groups.

The above results demonstrated that As^3+^ exposure alters the expression of CD71 and CD235a (Fig. 3a,b). The appearance or disappearance of CD71 and CD235a and their expression can be used to identify the phenotypes of erythroblasts, which reflect the degree of erythroid development stages^5,33,40,41^. For example, CD71^+^/CD235a^+^ cells are BasoE and PolyE, while the CD71^-/low^/CD235a^+^ subpopulations are OrthoE and ReticE^5,33,40,41^. In order to determine whether As^3+^ exposure affects the formation of each of these erythroblast subsets, we analyzed the distinct sub-populations of CD71 versus CD235a at the latest days (day 19 ± 1) of the time-course by flow cytometry, as shown in Fig. 4a. The subpopulations of BasoE and PolyE (CD71^+^/CD235a^+^) were increased in an As^3+^ dose-dependent manner (Fig. 4a,b). In contrast, the subpopulations of OrthoE and ReticE (CD71^−^/CD235a^+^), which are more differentiated erythroblasts, were decreased in a dose-dependent manner by As^3+^ exposure. The effects of As^3+^ on the erythroblast subsets (BasoE and PolyE, and OrthoE and ReticE) from three donors are summarized in Fig. 4b. The corresponding data for three individual donors is shown in Supplementary Fig. S8 (a-1, b-1 and c-1).

To further characterize erythroblast populations, we plotted CD235a^+^ cells using CD71 versus forward scatter (FCS), which further distinguish the populations of BasoE and PolyE, and OrthoE and ReticE^42,43^. The gated portion of CD235a^+^ cells with red rectangle (CD71^+^/CD235a^+^ and CD71^−^/CD235a^+^), as shown in Fig. 4a top-panel, were further divided into three subpopulations by plotting CD71 versus FCS as mentioned above. There was larger portion of BasoE (less mature cells) in the groups with As^3+^ addition than in control groups (Fig. 4a Bottom-panels). PolyE (the more mature cell population vs BasoE) was slightly decreased by 500 nM As^3+^ exposure as compared to control; however, OrthoE (the most mature cells investigated) was decreased by As^3+^ exposure in a dose-dependent manner (Fig. 4a Bottom-panels). The effect of As^3+^ exposure on the subpopulations of BasoE, PolyE and OrchoE based on the collective results from three donors is summarized in Fig. 4c, and the corresponding data from each individual donor is shown in Supplementary Fig. S8 (a-2, b-2, and c-2). These data further indicate that As^3+^ exposure inhibits differentiation and maturation of erythroblasts from the stages of BasoE to PolyE, and then to OrthoE.

### As^3+^ exposure causes decrease of Hb levels during the erythroid development of CD34^+^-HPCs

**Figure 5:**
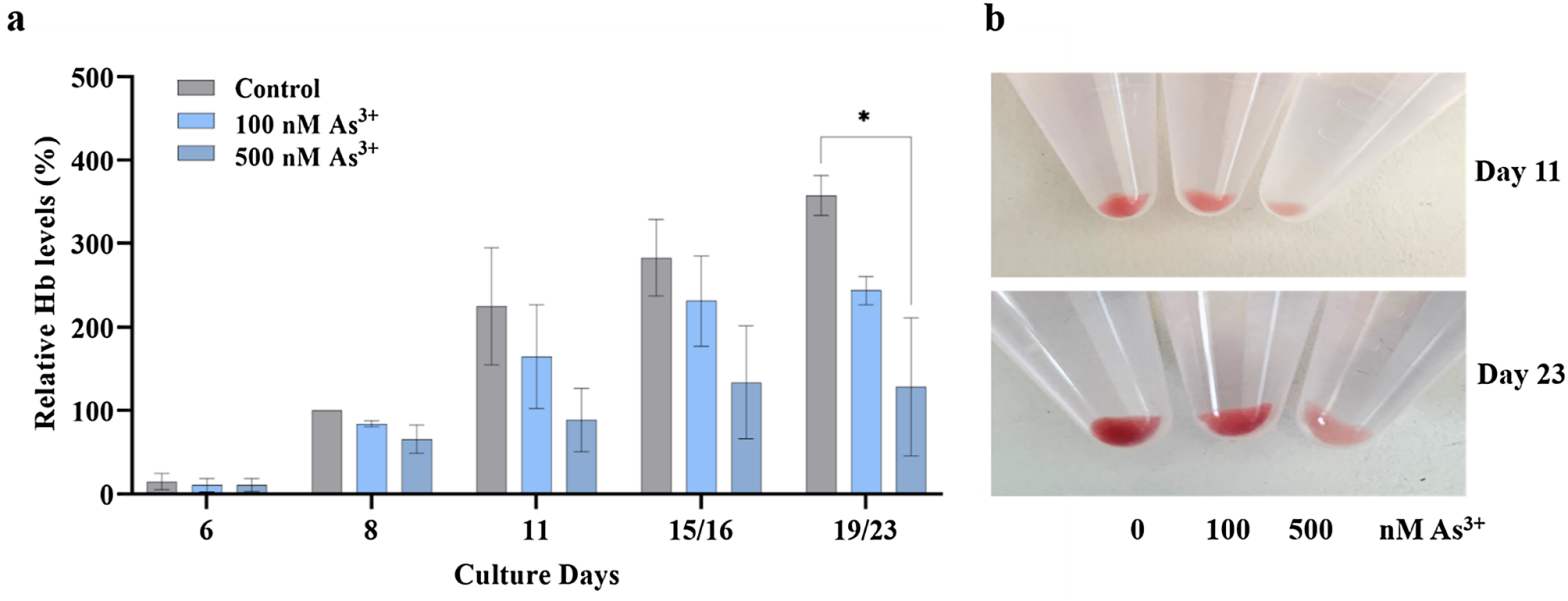
As^3+^ exposure decreases hemoglobin (Hb) production during the erythroid development of CD34^+^-HPCs. Human bone marrow CD34^+^-HPCs were cultured in erythroid expansion medium for 6, 8, 11, 15 or 16 (15/16) and 19 or 23 (19/23) days in the presence of 0, 100 nM, or 500 nM As^3+^. Cell pellets collected at the indicated time points were imaged, and then lysed for cytoplasmic protein extraction. The cell lysates with a total protein concentration of 100 ng/mL were used for quantification of Hb using the Human HBB SimpleStep ELISA Kit (ab235654, Abcam). (**a**) Relative Hb levels at the different time points tested. Relative Hb levels were calculated as described in the methods section. (**b**) Representative images of cell pellets from control, 100 nM, and 500 nM As^3+^ on days 11 and 23, which were from same donor (Donor 3). Data are expressed as summarized mean ± SD. *(*p* < 0.05) in one-way ANOVA, followed by Tukey’s post hoc test compared between groups.

Hb is the major product of erythropoiesis^1–4^. The production and accumulation of Hb starts from the committed erythroid progenitor stages of CFU-E onwards^38^. To examine the effect of As^3+^ exposure on Hb levels, we measured the expression of the beta subunit of Hb A, the major type of adult Hb, by ELISA, and compared the expression levels of Hb between control cells and cells exposed to As^3+^. The results show that relative Hb levels at each tested time points during erythroid expansion of CD34^+^-HPCs were reduced as a function of increasing of As^3+^ concentrations (Fig. 5a). The representative images of cell pellets, which were collected on days 11 and 23 of erythroid expansion, displayed visible red color changes due to As^3+^ exposure (Fig. 5b). These results suggest that Hb amounts were decreased by As^3+^ exposure in a dose-dependent manner at the time points tested (Fig. 5). The original ELISA and immunoblotting results from each of individual donors are shown in Supplementary Fig. S9.

## Discussion

Although murine models have provided useful insights on arsenic exposure mediated inhibition of erythropoiesis from in vitro and in vivo studies^11,12^; herein, we used human bone marrow hematopoietic progenitor CD34^+^ cells (CD34^+^-HPCs) as an in vitro model of erythropoiesis to investigate the effects of As^3+^ on human erythropoiesis. This is critical and essential because arsenic metabolism is different between mice and human^44^, and there is transcriptional divergence between human and mouse erythropoiesis^45,46^.

The present study demonstrated that As^3+^ exposure decreases the earliest stages of formation of committed erythroid progenitor differentiation, BFU-E and CFU-E, which suggests that As^3+^ exposure inhibits human erythropoiesis by decreasing the production of erythroid progenitors. This finding indicates that the effects of As^3+^ exposure on the differentiation of erythroid progenitors from humans and mice are similar^12,47^. We also demonstrated the similarity on the inhibitory effects of As^3+^ exposure on the generation of more differentiated erythroblasts between human and mice^12,47^. Importantly, the present study demonstrated that arsenic exposure leads to a reduction of hemoglobin content during the erythroid expansion of human CD34^+^-HPCs.

Since in normal erythropoiesis, the expression of the important cell surface antigens, including CD34, CD71, and CD235a, are tightly regulated^1,2^, alterations of these erythroid cell surface markers are a result of defective erythropoiesis. In this study, we individually tracked the dynamic changes of CD34, CD71, and CD235a during the erythroid expansion of human CD34^+^-HPCs, and compared their expression levels between control and As^3+^ exposed cells at each time point to understand how As^3+^ exposure affects human erythroid differentiation.

In erythropoiesis, CD34 is involved in enhancing proliferation and inhibiting differentiation^48,49^, the gradual declines of CD34 expression represent the initiation of erythroid development^5–7^. Our results in current study suggest that As^3+^ exposure may not affect the relevant molecules, which could interfere with the declines of CD34, because CD34 expression was not altered by As^3+^ exposure. However, As^3+^ exposure caused abnormal expressions of CD71 and CD235a during the erythroid expansion of human CD34^+^-HPCs, which could be the consequences of As^3+^-mediated dysregulations of the important molecular regulators of human erythropoiesis.

In the study of erythropoiesis, CD71 can be reliably used as an independent erythroid marker for immunohistochemical analysis of the bone marrow because it is selectively expressed at high levels in early erythroid progenitors and early erythroid precursors^7^. Moreover, CD71 is also as an excellent marker for erythroid disorder in bone marrow^6^, CD71 is significantly downregulated in dysfunctional erythropoiesis^50^, and is overexpressed in acute leukemia^51^. In current study, we found that As^3+^ exposure-mediated higher content of CD71 was throughout all time points investigated, especially in the late stages (longer culture days) of the erythroid expansion of CD34^+^-HPCs. Since the decline of CD71 at late stage of erythropoiesis is indispensable for differentiation and maturation of erythroblasts^5–7^, As^3+^-mediated unwanted increase of CD71 signal at late stage of erythroid development may be a sign of the inhibition or erythroblasts differentiation.

Moreover, CD71, the transferrin receptor, mediates the uptake of transferrin-iron complexes^52,53^ and is essential for Hb synthesis^54^ during the differentiation of erythroid progenitors (BFU-E, CFU-E) and early erythroblasts^1–3^. CD71 is highly expressed in CFU-E cells during normal erythropoiesis^5–7^. We found that As^3+^ reduced the formation of BFU-E and CFU-E, but CD71 expression increases rather than decreases. Therefore, the As^3+^-exposure-mediated increase of CD71 signal and decrease of BFU-E and CFU-E in the early stages of erythroid differentiation is somewhat contradictory. The observed phenomena in this study suggests an abnormal molecular regulation associated with As^3+^-exposure and needs to be further investigated.

CD235a is the erythroid specific marker and is expressed in early erythroid precursors, and highly expressed in reticulocytes and erythrocytes. The increased expression of CD235a is indispensable for normal differentiation and maturation of erythroblasts^5–7^. Our data showed that exposure to 500 nM As^3+^ resulted in the decrease of CD235a signal at the later points of the time course investigated. This suggests that the decrease of more differentiated erythroblasts may not only caused by the upstream inhibition of erythroid progenitors (BFU-E and CFU-E), but also by impairing the terminal erythroid differentiation of erythroblasts at the late stage of erythropoiesis.

Erythropoiesis is a stepwise continuing processes^1,2^. When exposed to As^3+^, the impact of As^3+^ may not be limited to early-stage progenitors (BFU-E and CFU-E). In order to determine whether As^3+^ exposure directly impairs the differentiation of erythroblasts, additional studies are needed. However, our findings that As^3+^ mediates the abnormal expression of CD71 and CD235a during erythroid expansion, may provide useful information for further investigations on the molecular mechanism by which As^3+^ exposure impairs multiple stages of erythropoiesis and causes anemia.

Anemia can be caused by either decreased generation of RBC or reduced production of hemoglobin or both^28–30^. The level of Hb is an important criterion used clinically for identifying dysfunctional erythropoiesis or anemia. Zhang, et al. reports that arsenic trioxide (ATO) significantly decreased the mRNA expression levels of *HBA*, *HBB*, and *HBG* in K562 cells, which is a human erythroleukemia cell line^55^. In the present study, we demonstrated that As^3+^ exposure causes reduction of hemoglobin, which further demonstrating the inhibitory effect of low-dose As^3+^ on human erythropoiesis.

CD71 is required for iron delivery from transferrin to immature erythroblasts^52,53^. Taking into consideration that iron is the indispensable component of heme, which is the important iron-containing porphyrin compound of the globin chains of Hb^54^. Our data show that As^3+^ exposure increases expression of CD71, but decreases Hb levels. These observations imply that the As^3+^ exposure-induced reduction of Hb may not be associated with the increased CD71 in our experimental model system. Observations were reported in a cross-sectional study of anemia and iron deficiency as risk factors for arsenic-induced skin lesions in Bangladeshi women, which showed that 75% of women with anemia had adequate iron stores suggesting that the majority of anemia detected in this arsenic-exposed population was unrelated to iron depletion^23^.

Regarding the As^3+^-exposure mediated reduction of Hb, there are two primary ways this may be caused. One is an indirect mechanism that is dependent on decreased numbers of erythroid progenitors (BFU-E/CFU-E) as well as reduced numbers of later-stage erythroblast subsets. Our data suggest this may be the factor responsible for As^3+^-mediated reduction of Hb. However, another possible mechanism is that As^3+^ interferes with Hb synthesis directly. This is an especially important consideration since our previous report shows that As^3+^ interacts with the zinc finger motifs of GATA-1, resulting in the dysfunction of this key transcription factor^47^. GATA-1 is of the most important regulators of erythropoiesis, controlling not only erythroid proliferation and differentiation but also the production of Hb^56,57^. Therefore, although further investigations of how As^3+^ exposure reduces the formation of Hb are needed, dysfunction of GATA1 may be a significant contributing factor to the As^3+^ exposure-mediated reduction of Hb observed in this study.

We found that As^3+^ exposure leads to the inhibition of cell survival in the later-stages of erythroid development. This finding suggests that As^3+^ exposure causes an inhibition of erythropoiesis by influencing multiple biological functions and processes such as cell death. A recent report from our laboratory showed that apoptotic cell death is an important mechanism involved in the loss of erythropoietic output in mice^58^. Concerning the strong inhibitory effect of As^3+^ on BFU-E/CFU-E and on cell growth during the erythroid expansion of CD34^+^-HPCs, cell death may also be a critical factor in the loss of early erythroid progenitors and late-stage erythroblasts. This requires further studies focused on understanding how arsenic exposure affects the formation of RBCs during erythropoiesis.

In conclusion, low dose As^3+^ exposure may inhibit erythropoiesis by impairing multiple stages of differentiation, including the generation of erythroid progenitor cells, differentiation of erythroblasts, and possibly also by influencing multiple biological functions and process including proliferation, cell survival, and Hb production. This study characterized the overall effects associated with arsenic-induced inhibition of erythropoiesis using a human-relevant in vitro model of erythropoiesis. Such findings are critical for further studies on the molecular mechanisms by which arsenic interferes with erythropoiesis and causes anemia in humans.

## Materials and methods

### Human bone marrow CD34^+^ cells

Human adult bone marrow CD34^+^ cells are multipotential HPCs, which can develop to RBCs under ex vivo culture condition using erythroid specific growth factors and hormones^31–37^.

In this study, human bone marrow CD34^+^-HPCs (Cat. #70002), the culture medium (StemSpan SFEM II Medium, Cat. #09655), and the supplements (StemSpan Erythroid Expansion Supplement, 100X, Cat. #02692), which includes all of necessary growth factors, cytokines and hormones (such as IL-3, SCF, TPO and EPO) for erythroid expansion and differentiation, were purchased from STEMCELL Technologies.

To confirm that our findings and observations are not individual donor-related phenomena, CD34^+^-HPCs from three individual donors (Donor 1, Donor 2, and Donor 3) were used independently in all the experiments as described below. We present the statistical results of the three donors in the text. The results of each donor individually (Donor 1, 2 and 3) are provided in the Supplemental Materials.

### Arsenic exposure

Sodium (meta) arsenite (NaAsO_2_, ≥ 95% purity, CAS 774–46-5; Millipore Sigma, Cat. #S7400) was used throughout this study. As^3+^ stock solutions of 100 mM were prepared in 1× PBS (pH7.4), aliquoted to 20 µL/tube after sterile filtration and stored at − 20 °C prior to use. This stock solution was diluted to the required concentrations immediately with culture medium before usage. To ensure consistent As^3+^ exposure levels throughout the duration of our experiments^59^, we replaced the culture medium with fresh media containing As^3+^ every 2–3 days during the time course investigated.

Since the WHO safety standard for arsenic in drinking water is ≤ 10 µg/L^12^, two final concentrations at 100 nM (12.991 µg/L) and 500 nM (64.955 µg/L) of As^3+^ were used in the present experiments to test the effect of low dose As^3+^ exposure on human erythropoiesis. Although these doses are higher than the WHO safety standard, they represent environmentally relevant levels that are commonly experienced by human populations^12,14^.

### Erythroid expansion or erythroid differentiation of human bone marrow CD34^+^-HPCs

CD34^+^-HPCs at the initial number of 10,000 cells per mL were plated in StemSpan SFEM II medium supplemented with the Erythroid Expansion Supplement, as well as with the addition of 0, 100 nM or 500 nM of As^3+^. The CD34^+^-HPCs were maintained at 37 °C in a cell culture incubator with a humidified atmosphere of 95% and 5% CO_2_. Fresh medium with supplements and As^3+^ were replaced every 2–3 days throughout the duration of the experiment.

### Measurement of CD34^+^-HPCs proliferation during erythroid expansion and differentiation

Total cell numbers were counted using a hemocytometer at multiple time points of CD34^+^-HPCs erythroid expansion: days 2, 4, 6, 8 (+ 1), 11, 15 (+ 1), 19 (± 1). The variations in days were due to practical concern and feasibility. For example, days 8 (+ 1) indicates that the experiment was performed on day 8 for some donor, but for rest donors, the experiments were carried out on day 9. Briefly, the cell suspension was mixed with an equal volume of trypan blue and 20 μL of the mixture was loaded onto a hemocytometer. Cells were counted in the 4 outer squares of the grid layout and the cell concentration was calculated and expressed as total cell number per mL.

### Colony-Forming Unit (CFU) assays of human bone marrow CD34^+^-HPCs

Colony-Forming Unit (CFU) assays were conducted using the MethtoCult system from STEMCELL Technologies (MethtoCult-H44335 enriched). This method has been validated for studying human hematopoietic progenitor cell differentiation^60,61^. This assay was performed according to the manufacturer instructions. Briefly, three tubes of CD34^+^-HPCs suspensions were prepared at 5 × 10^3^ cells per mL, then As^3+^ was added into each tube to yield CD34^+^-HPCs suspensions containing 0, 100 nM or 500 nM of As^3+^. The cells/As^3+^ mix (0.5 mL) was added separately to 3 aliquots of 5 mL MethoCult, and mixed thoroughly to make 5.5 mL of MethoCult containing 500 CD34^+^-HPCs per mL. This mixture was then dispensed separately 1.1 mL into four non-treated 35 mm culture dishes (*n* = 4 technical replicates). The plates were incubated at 37 °C in 5% CO_2_, ≥ 95% humidity for at least 16 days. After the incubation period, the resulting BFU-E and CFU-E colonies were identified (i.e., based on morphology and color of the colonies) and counted using an inverted light microscope at days 14 and 16, respectively.

The common standards used to evaluate the capability of proliferation and differentiation of erythroid progenitors is to measure the ratios of BFU-E or CFU-E numbers to the initial input cell number^62^. For example, when 500 human CD34^+^-HPCs are mixed with erythroid supportive medium, which is suitable for erythroid lineage differentiation, 120 BFU-E and 250 CFU-E colonies appeared. Therefore, the ratio of BFU-E and CFU-E colonies derived from CD34^+^-HPCs is 120/500 and 250/500, respectively.

Concerning the variation in individual donor-response to the As^3+^ exposures, we converted the numbers of BFU-E and CFU-E colonies to a relative number in order to summarize the results of BFU-E and CFU-E from three different donors for statistical analysis. We defined the original numbers of BFU-E and CFU-E colonies of control cells (BFU-E^control^ or CFU-E^Control^) as 100, then the number of BFU-E or CFU-E colonies of each of samples (BFU-E^Sample^ or CFU-E^Sample^) was converted to relative numbers by the formulas: BFU-E^Sample^/BFU-E^Control^ × 100, or CFU-E^Sample^/CFU-E^Control^ × 100.

### Analysis of cell surface marker by flow cytometry (FCM)

Flow cytometric analysis of the cell surface markers, CD34, CD235a and CD71, was conducted at each culture time point: days 2, 4, 6, 8 (+ 1), 11, 15 (+ 1), 19 (± 1). Briefly, 1 × 10^5^ cells from control and each exposure group were pelleted by centrifugation at 1000 rpm. Following two washes with flow cytometry wash buffer (1× PBS and 2% Heat-inactivated Fetal Bovine Serum (HI-FBS)), cells were co-labeled with BV421-conjugated anti-human CD34 (BD Pharmingen, Cat. #562577), APC-conjugated anti-human CD71 (BD Pharmingen, Cat. #551374), and PE-conjugated anti-Human CD253a (BD Pharmingen, Cat.# 561051) for 30 min at 4 °C with gentle mixing. The isotype controls included in this analysis were BV421 Mouse IgG_1_, κ (BD Pharmingen, cat. #562438), APC Mouse IgG2a k Isotype Control (BD Pharmingen, Cat. #555576) and PE Mouse IgG2b κ Isotype Control RUO (BD Pharmingen, cat. # 555743). Single antibody staining and compensation controls were used in all experiments.

Following surface marker staining, cells were stained with Zombie NIR (BioLegend, Cat. #423105) to distinguish live and dead cells. Stained cells were analyzed for the progenitor cell surface marker CD34 (CD34-BV421) and erythroblast surface markers CD71 (CD71-APC) and CD253a (CD235a-PE) using the AttuneNxT Acoustic Focusing Flow Cytometer (Life Technologies).

The geometric mean intensity of CD71-APC and CD235a-PE were summarized as the relative numbers due to the variation in individual donor-response to the As^3+^ exposures, and some variation caused by staining and acquiring conditions et al. We defined each original value of mean intensity of control cells (Mean^Control^) as 100, then the original mean intensity of each of sample (Mean^Sample^) was converted to relative numbers by the formulas: Mean^Sample^/Mean^Control^ × 100.

### Quantification of Hb levels

At each time point of erythroid differentiation, cells from control and each exposure group were collected and lysed for cytoplasmic protein extraction. Cell lysates were evaluated for total protein concentration using Pierce 660 nm Protein Assay Kit (Cat. # 22662, Thermo Fisher Scientific). Hb level in cell lysates with a total protein of 100 ng/ml was measured using Human HBB SimpleStep ELISA Kit (ab235654, Abcam). The cell lysates at each time points were diluted to an appropriate protein concentration to meet the measurement range of the kit. The ELISA measurement was conducted following the instructions and protocol provided by the manufacturer.

Concerning the variation in individual donor-responses to the As^3+^ exposure, in order to summarize the data from 3 donors, we defined the level of Hb in control cells at day 8 (Hb^Control_Day 8^) as 100 (%), while the relative level of Hb of each of other samples (Hb^Sample^) at different time points were calculated by [(Hb^Sample^/Hb^Control_Day 8^) × 100].

### Statistics

Repeated measurements on the experiments with CD34^+^-HPCs from the three bone marrow donors were taken at the indicated time points of each experiment (*n* = 3). Each experiment was repeated (at least 2-times) to account for the technical variability. Data were summarized using mean ± SD. Differences among the groups were determined using one-way repeated measures ANOVA, followed by pairwise comparison using the Tukey’s post hoc test. Statistical significance was considered at *p* < 0.05 (*), < 0.01 (**), < 0.001 (***), or < 0.0001 (****), as indicated in figures and figure legends. All analyses were performed with GraphPad Prism 7.

## Supplementary Information

Supplementary Figures.
