# Supplementary material for: Arsenite exposure inhibits the erythroid differentiation of human hematopoietic progenitor CD34+ cells and causes decreased levels of hemoglobin
Source: Sci Rep. 2021 Nov 11;11:22121. doi: 10.1038/s41598-021-01643-2 (PMC8586241; doi:10.1038/s41598-021-01643-2)
Supplement: Supplementary file 1 — Supplementary Figures. [file 41598_2021_1643_MOESM1_ESM.docx]

**Supplementary material**

**Arsenite Exposure Inhibits the Erythroid Differentiation of Human Hematopoietic Progenitor CD34+ Cells and Causes Decreased Levels of Hemoglobin**

Guanghua Wan^1^, Sebastian Medina^1,2^, Haikun Zhang^1^, Rong Pan^1^, Xixi Zhou^1^, Alicia M Bolt^1^, Li Luo^3^, Scott W. Burchiel^1^ and Ke Jian Liu^1*^

^1^Department of Pharmaceutical Sciences, The University of New Mexico College of Pharmacy, Albuquerque, NM, 87131, United States

^2^Department of Biology, New Mexico Highlands University, Las Vegas, NM 87701, United States

^3^Division of Epidemiology, Biostatistics and Preventive Medicine at the University of New Mexico, Albuquerque, NM, 87131, United States

*To whom correspondence should be addressed: Ke Jian Liu, Research Incubation Facility, The University of New Mexico College of Pharmacy, Department of Pharmaceutical Sciences, Albuquerque, NM 87131, United States. Email: [KLiu@salud.unm.edu](mailto:KLiu@salud.unm.edu). Phone: 505 272-9546. Fax: 505 272-0704

**Supplementary Figure S1**

**
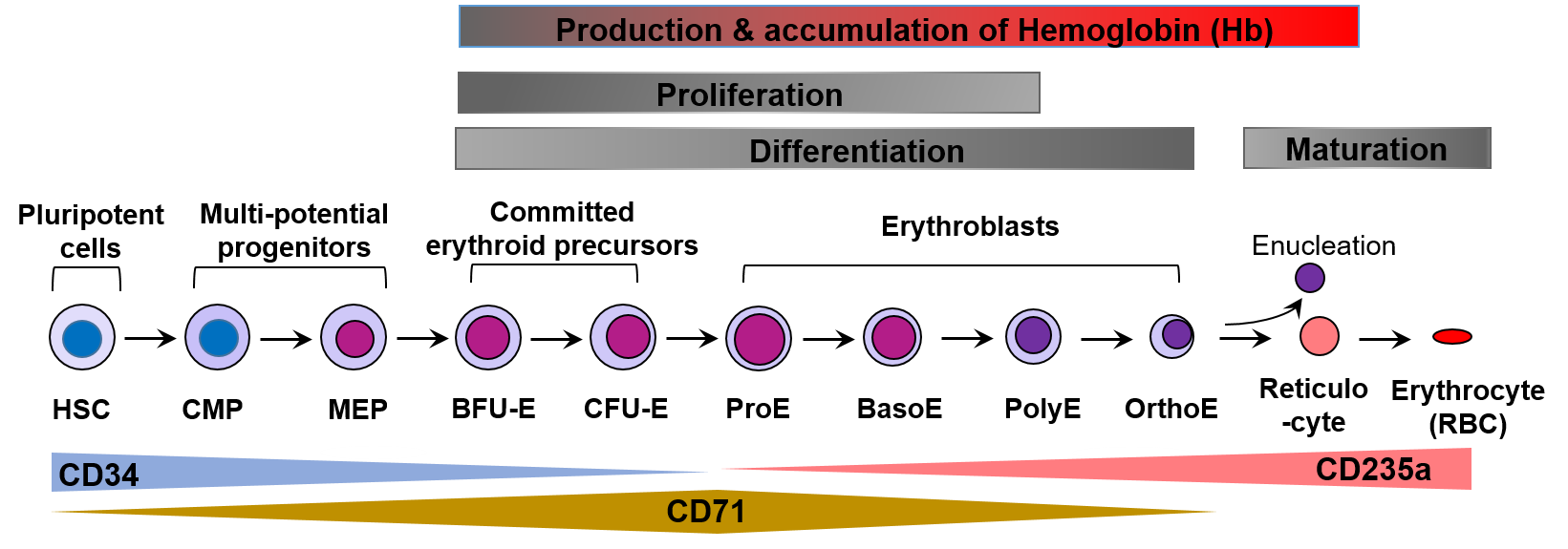
**

**Supplementary Fig. S1. Schematic diagram of normal red blood cell (RBC) production.**

**Supplementary Figure S2**


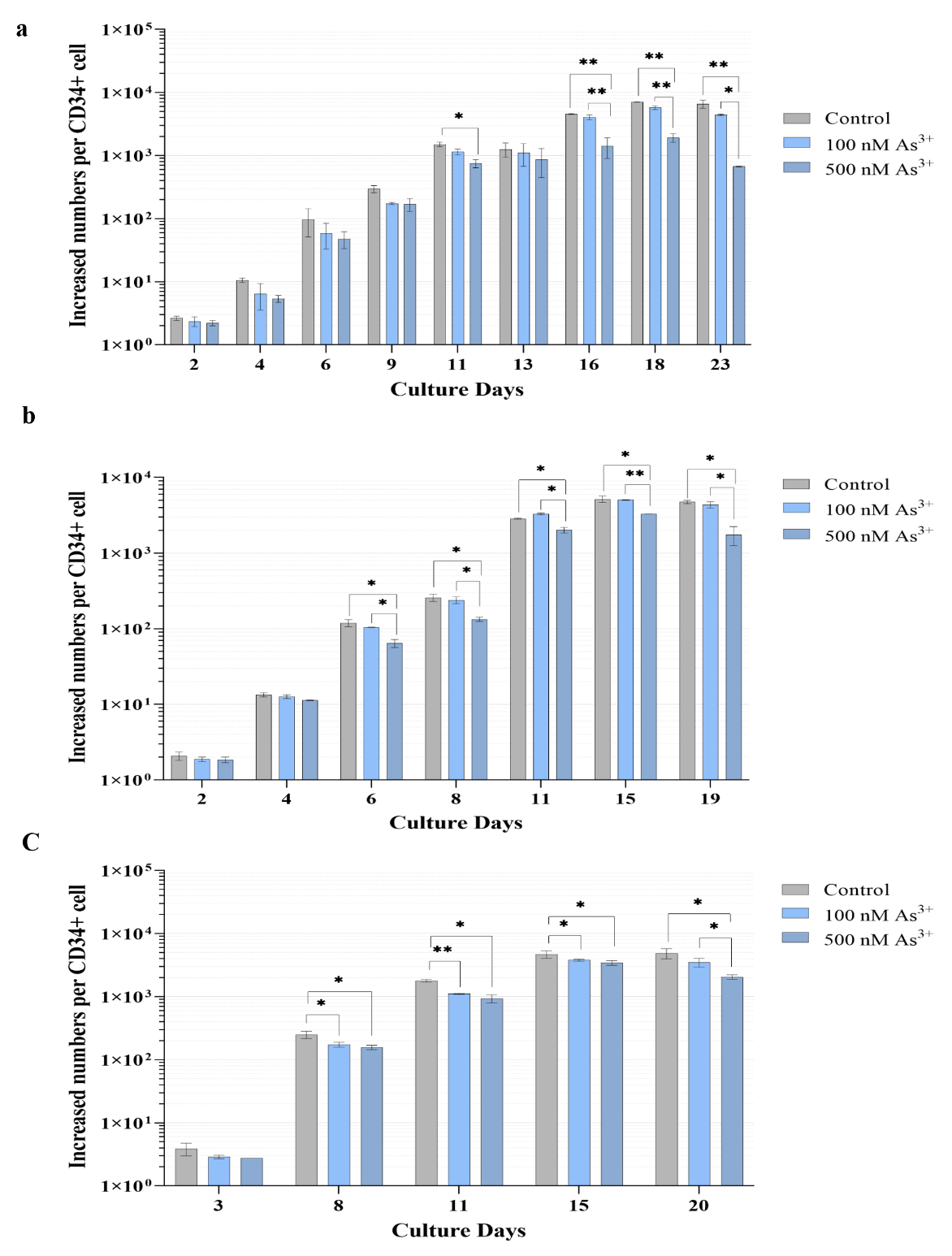


**Supplementary Fig. S2.** **Inhibition of cells growth by As^3+^ exposure during erythroid expansion of CD34^+^-HPCs.** Human Bone Marrow CD34^+^-HPCs at an initial concentration of 1×10^4^ cells/mL were cultured for the indicated days in erythroid expansion medium with or without 100 or 500 nM As^3+^. At each indicated time point, cell growth was recorded as the counts per initial CD34^+^-HPC, which come from (**a**) Donor 1, (**b**) Donor 2, and (**c**) Donor 3. Data are expressed as mean ± SD, *n* = 2 technical repeats/group, * (*p*<0.05), and ** (*p*<0.01) in one-way ANOVA, followed by Tukey’s post hoc test between groups at indicated time points.

**
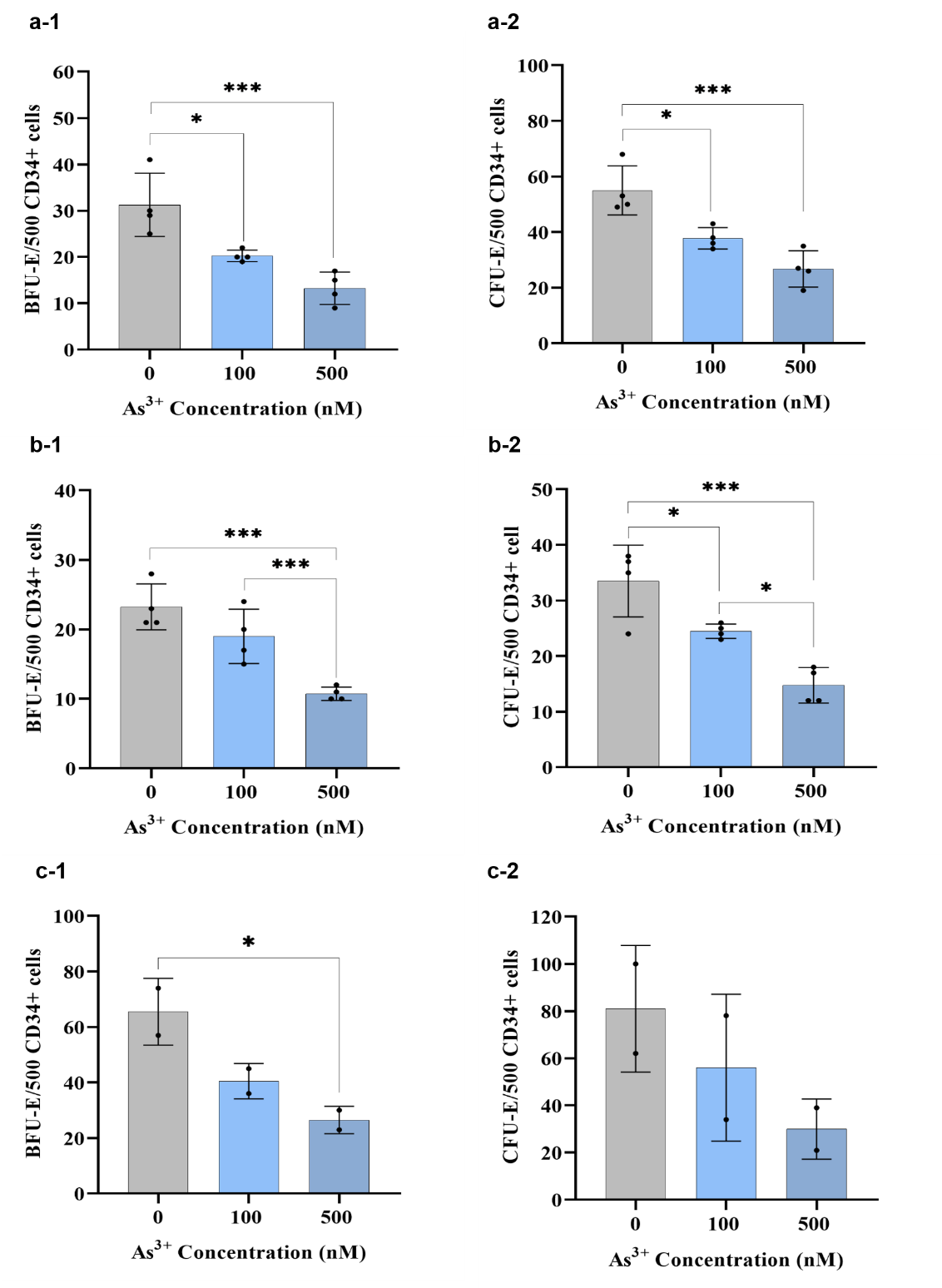
Supplementary Figure S3**

**Supplementary Fig. S3.** **As^3+^ exposure suppresses BFU-E and CFU-E formation (individual results from 3 Donors).** Five-hundred Human Bone Marrow CD34+ cells were mixed with 1.1 ml of MethoCult^TM^ medium containing 0, 100 nM, or 500 nM As^3+^. After 14-16 days of incubation, colonies were scored under an inverted light microscope. Numbers of BFU-E and CFU-E colonies are **(a)** for Dorner 1, **(b)** for Dorner 2, and **(c)** for Donor 3. Data are expressed as mean ± SD, *n* = 4 (Donor 1 and 2) or *n* = 2 (Donor 3), for technical repeats/group, * (*p*<0.05), ** (*p*<0.01), and *** (*p*<0.001) in one-way ANOVA, followed by Tukey’s post hoc test between groups.

**Supplementary Figure S4**

**
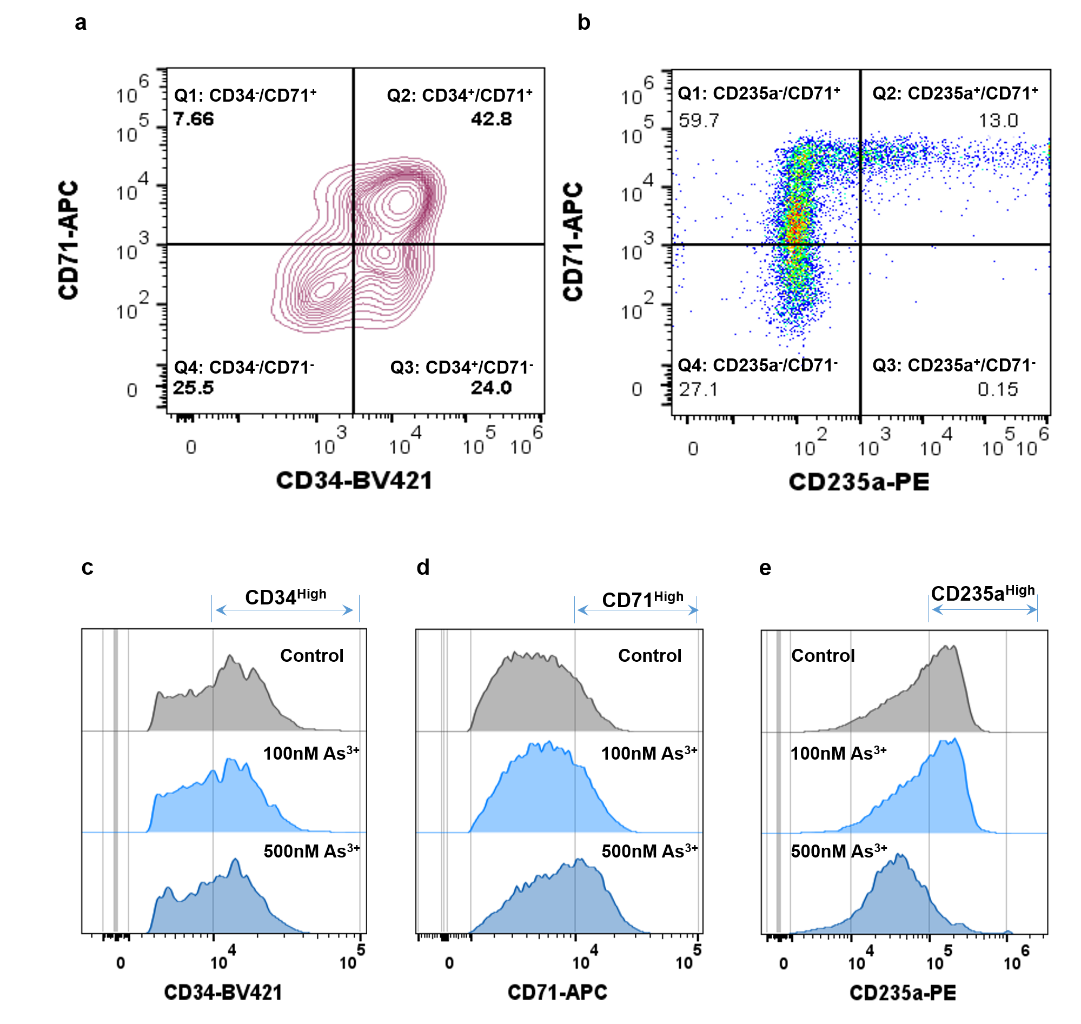
**

**Supplementary Fig. S4. Methods for determining cell subpopulations: CD34^+^ and CD34^High^, CD71^+^ and CD71^High^, as well as CD235a^+^ and CD235a^High^.** Human bone marrow CD34^+^-HPCs (1×10^4^ cells/ml) were cultured for 2-20 days in erythroid expansion medium with the addition of 0 (Control), or 100 or 500nM As3+. At each time points, cells were stained with BV421-conjugated antibody against CD34 (CD34-BV421), APC-conjugated antibody against CD71 (CD71-APC), and PE-conjugated antibody against CD235a. Flow cytometry was followed. **(a)** Method for defining CD34 positive (CD34^+^) and CD71 positive (CD71^+^) cell populations by plot of CD71-APC versus CD34-BV421. CD34^+^ cells include two subpopulations CD34^+^/CD71^+^ (Q2) and CD34^+^/CD71^-^ (Q3), while CD71^+^ cells are the sum of two subpopulations CD34^-^/CD71^+^ (Q1) and CD34^+^/CD71^+^ (Q2), respectively. **(b)** Plot of CD71-APC versus CD235a-PE shows the defining method of CD235a positive (CD235a^+^) cell population, which includes two subpopulations CD235a^+^/CD71^+^ (Q2) and CD235a^+^/CD71^-^ (Q3). **(c)** The definition of CD34^High^ population, which is based on the fluorescence intensity of CD34-BV421 in a histogram of CD34^+^ population. The abscissa of histogram measures the fluorescence (BV421) intensity of CD34^+^ cells, while the ordinate gives the cell counts corresponding to the fluorescence intensity. CD34^High^ are the cells with CD34-BV421 intensity bigger than 10^4^. **(d)** Define methods of CD71^High^, in the Histograms of total CD71^+^ cells, the fluorescence intensity > 10^4^ was defined as CD71^High^. **(e)** Examples for analysis of CD235a expression level based on the intensity of CD235a-PE. The abscissa of histogram measures the fluorescence (PE) intensity of CD235a^+^ cells, while the ordinate give the cell counts corresponding to the fluorescence intensity of CD235a-PE. CD235a^High^ are defined as the intensity of CD235a-PE bigger than 10^5^.

**Supplementary Figure S5**

**
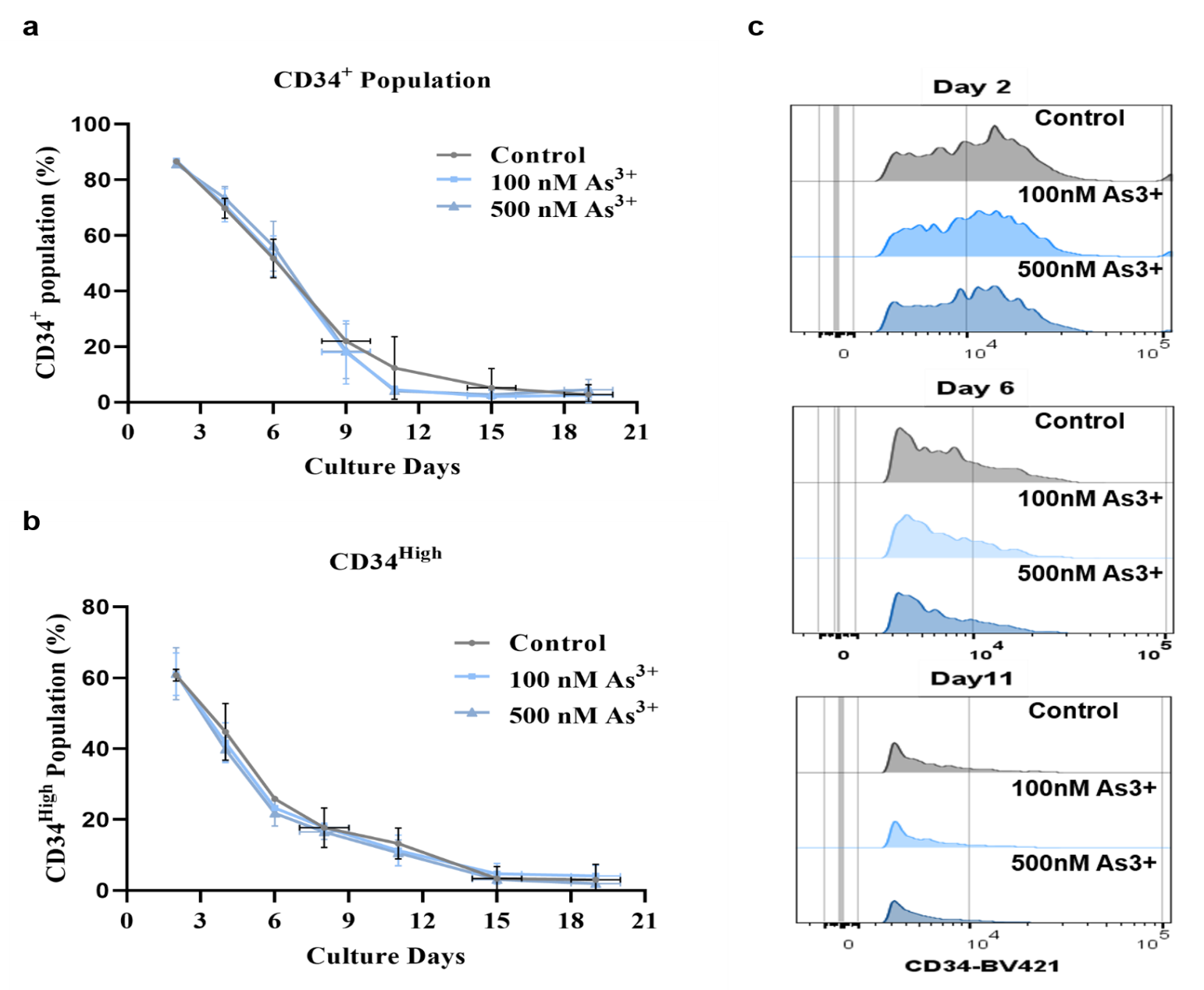
**

**Supplementary Fig. S5. As^3+^ exposure did not alters the dynamic changes of CD34^+^ population and CD34^High^ proportion during erythroid development of CD34+ cells.** Human bone marrow CD34^+^-HPCs (1×10^4^ cells/ml) were cultured for 2-19 (±1) days in erythroid expansion medium with the addition of 0, or 100nM or 500nM As3+. At the time points (days 2, 4, 6, 8/9, 11, 15/16, and 18/19/20), cells were stained with BV421-conjugated antibody against CD34 (CD34-BV421). Flow cytometry analysis was followed. The definition of each cell population, including CD34^+^ and CD34^High^ were indicated on Supplementary Figure 3 (S3). **(a)** Dynamic change curves of total CD34^+^ population through over the time course. **(b)** The dynamic change curve of CD34^High^ along the extension of cell culture, which displays that the expression level of CD34 protein decreased accompanying with erythroid development. **(c)** The histogram of CD34 show the decline of CD34^+^ cells and CD34^High^ cells. Data are expressed as summarized mean ± SD from three donors, *n* = 3 repeats/group, no significance in one-way ANOVA, followed by Tukey’s post hoc test compared between the groups.

**Supplementary Figure S6**

**
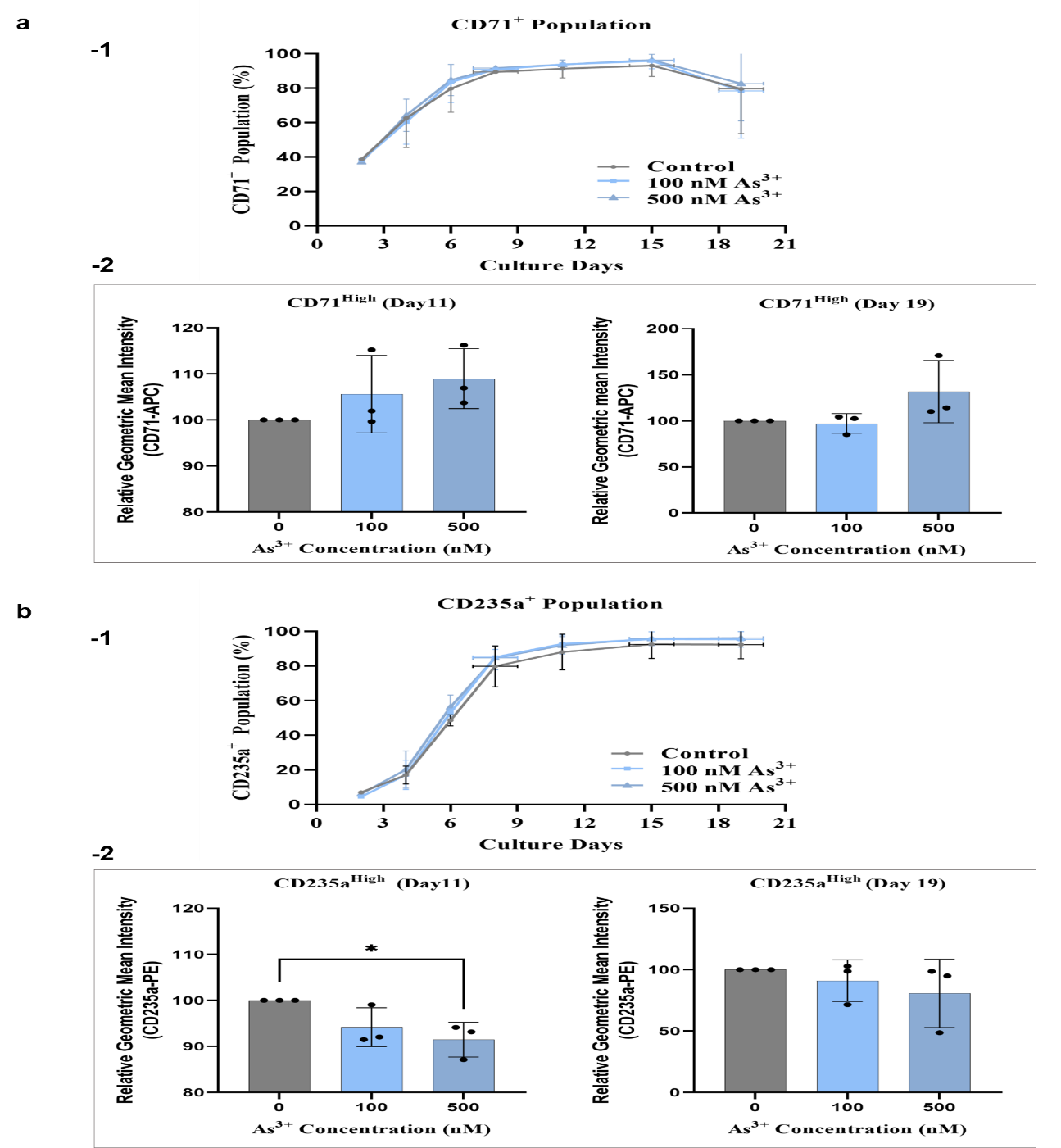
**

**Supplementary Fig. S6. As^3+^ exposure increases CD71 signal but decreases CD235a level during erythroid expansion of CD34+ cells.** Human bone marrow CD34^+^-HPCs (1×10^4^ cells/ml) were cultured for the indicated days in erythroid expansion medium with or without As^3+^. At each indicated time point, cells were stained with APC-conjugated antibody against CD71 (CD71-APC), and PE-conjugated antibody against CD235a. Flow cytometry was followed. The definition of CD71^+^, CD71^High^, CD235a^+^, and CD235a^High^ were indicated on Supplementary Fig. S3. (**a-1**) CD71^+^ population changes cross the time-course investigated. (**a-2**) As^3+^ exposure affect geometric mean intensity of CD71^High^ on days 11 and 19. (**b-1**) CD235a^+^ population changes cross the time-course investigated. (**b-2**) As^3+^ exposure decreases geometric mean intensity of CD235a^High^ on days 11. The * or *** indicates the significance of 100 nM or 500 nM As^3+^ vs Control. Data are expressed as mean ± SD, *n* = 3 repeats/group (3 donors), * (*p*<0.05), ** (*p*<0.01), and *** (*p*<0.001) in one-way ANOVA, followed by Tukey’s post hoc test between the groups.

**Supplementary Figure S7**

**
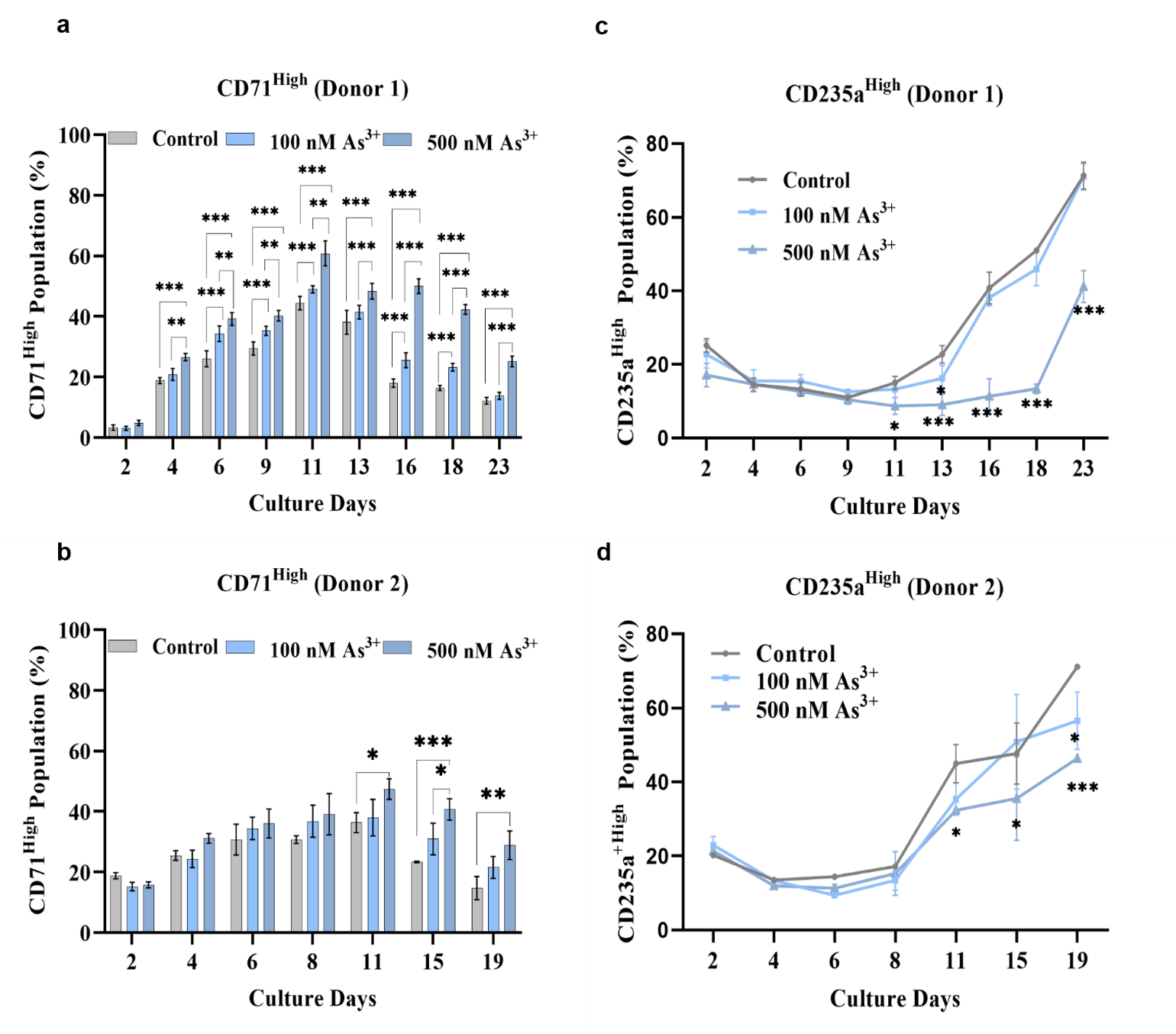
**

**Supplementary Fig. S7. As^3+^ exposure alters the dynamic change of CD71^High^ and CD235a ^High^ expression during erythroid development of CD34^+^-HPCs (Donor 1 and 2).** Human bone marrow CD34^+^-HPCs (1×10^4^ cells/ml) were cultured for the indicated days in erythroid expansion medium with or without As^3+^. At each indicated time point, cells were stained with APC-conjugated antibody against CD71 (CD71-APC), and PE-conjugated antibody against CD235a. Flow cytometry was followed. The definition of CD71^High^, and CD235a^High^ were indicated on Supplementary Fig. S3. **(a), (b)** As^3+^ exposure increases CD71^High^ populations though over the time course investigated for donor 1 and 2. **(c), (d)** As^3+^ (500nM) exposure decreases CD235a^High^ at late stage for donor 1 and 2. The * or *** indicates the significance of 100 nM or 500 nM As^3+^ vs Control. Data are expressed as mean ± SD, *n* = 2 repeats/group, * (*p*<0.05), ** (*p*<0.01), and *** (*p*<0.001) in one-way ANOVA, followed by Tukey’s post hoc test between the groups.

**Supplementary Figure S8**

**
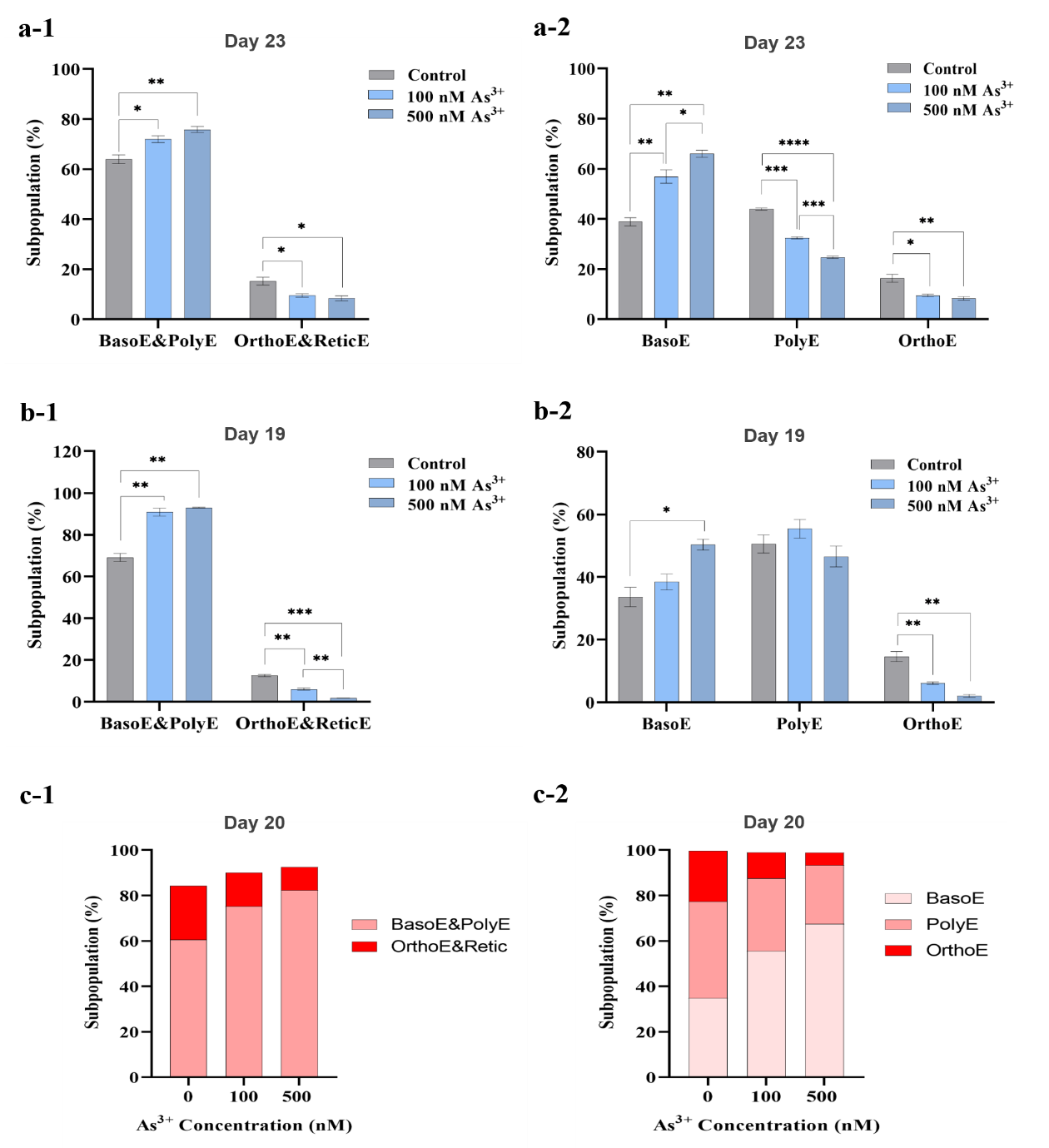
**

**Supplementary Fig. S8. As^3+^ exposure inhibits erythroblast differentiation and maturation at the latest day investigated of erythroid development of CD34^+^-HPCs (individual results from 3 Donors).** Human bone marrow CD34^+^-HPCs were incubated in erythroid expansion medium with or without As^3+^ for 23 days (Donor 1), 19 days (Donor 2), or 20 days (Donor 3). Flow cytometry was followed. (**a-1**) & (**b-1**) & (**c-1**) As^3+^ exposure increases the population of less mature erythroblasts (BasoE/PolyE) and decreases the number of more mature erythroblasts (OrthoE/ReticE). These different erythroblast phenotypes were identified based on the plot of CD235a versus CD71 as showed in Fig. 3a top-panel. (**a-2**) & (**b-2**) & (**c-2**) As^3+^ exposure increases the population of less mature erythroblasts, BasoE and decreases the number of more mature erythroblasts, OrthoE. These erythroblasts were identified from CD235a^+^ population based on the plot of CD71 versus FSC (Fig.3a bottom-panel). Data are expressed as mean ± SD, *n* = 2 for technical repeats/group, * (*p*<0.05), ** (*p*<0.01), and *** (*p*<0.001) in one-way ANOVA, followed by Tukey’s post hoc test between groups.

**Supplementary Figure S9**

**
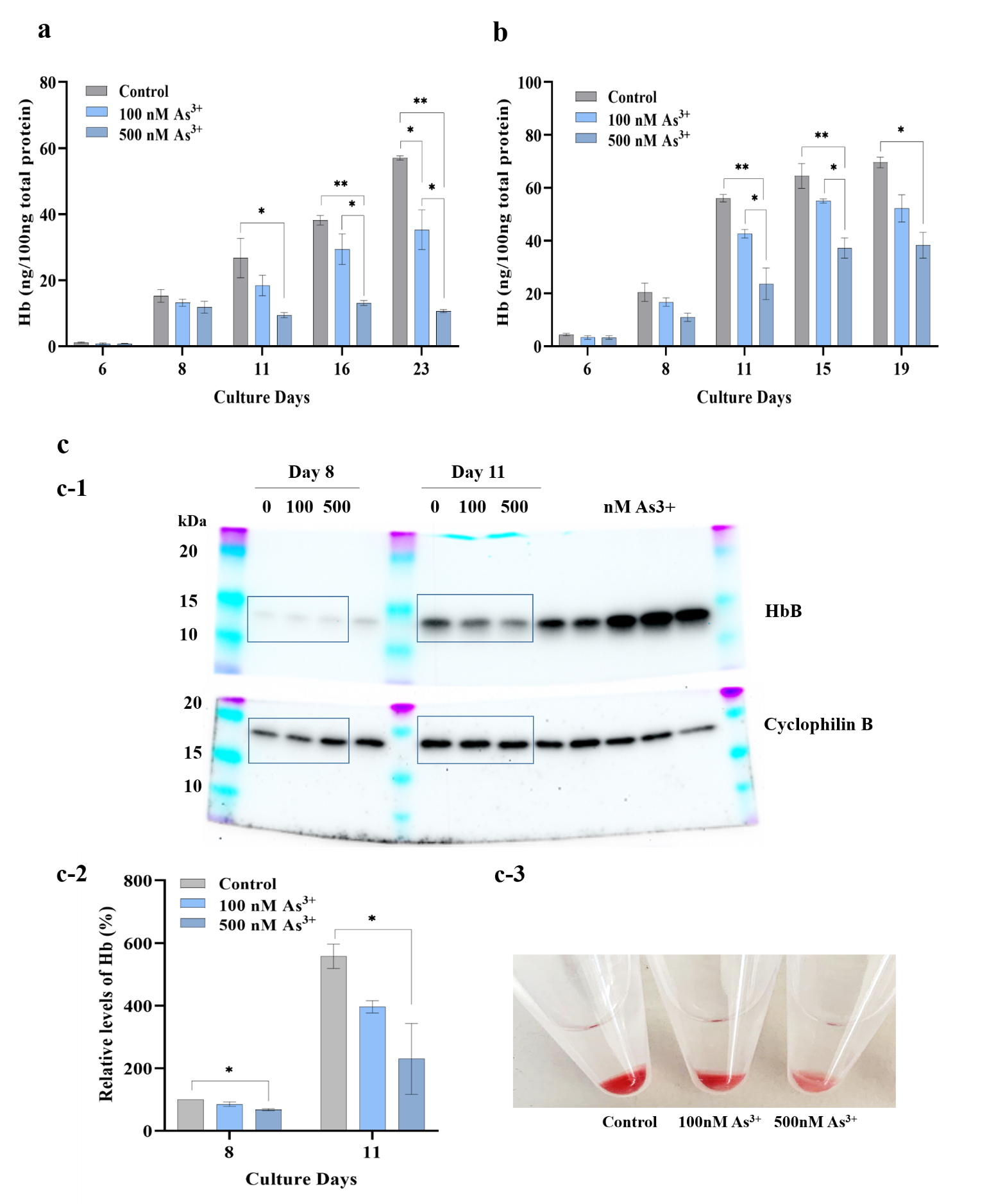
**

**Supplementary Fig. S9. As^3+^ exposure decreases Hb production during the erythroid development of CD34^+^-HPCs (individual results from 3 Donors).** Human Bone Marrow CD34^+^-HPCs were cultured in the medium for erythroid expansion at the presence of 0 (Control), 100 nM, or 500 nM As^3+^. Cell pellets collected at the indicated time points were imaged, and then lysed for cytoplasmic protein extraction. The cell lysates with a total protein concentration of 100 ng/mL were used for quantification of Hb using the Human HBB SimpleStep ELISA Kit (ab235654, Abcam) or immunoblotting analysis. (**a**) Hb levels at the indicated time points by ELISA measurement (Donor 1). (**b**) Hb levels at the indicated time points by ELISA measurement (Donor 2). **(c)** Hb levels at the indicated time points by immunoblotting quantification, and images of cell pellets on day 11 (Donor 3). (**c-1**) representative images of immunoblotting. Each 1µg total protein was loaded onto a SDS-PAGE gel and transferred to a membrane. Hb on the membrane was detected by human anti-HbB antibody (Cat. # ab214049, Abcam). The relative amount of Hb was estimated by densitometer based on the intensity of immunoblotting bands. (**c-2**) the summary of duplicate immunoblotting analysis of Hb. (**c-3**) images of cell pellets of control, 100nM As^3+^ and 500nM As^3+^ on day 11. Data are expressed as mean ± SD, *n* = 2 for repeats/group, * (*p*<0.05), and ** (*p*<0.01) in one-way ANOVA, followed by Tukey’s post hoc test between groups.
